# The Functional Trajectory in Frail Compared With Non-frail Critically Ill Patients During the Hospital Stay

**DOI:** 10.3389/fmed.2021.748812

**Published:** 2021-11-04

**Authors:** K. E. Fuest, Marco Lorenz, Julius J. Grunow, Björn Weiss, Rudolf Mörgeli, Sebastian Finkenzeller, Ralph Bogdanski, Markus Heim, Barbara Kapfer, Silja Kriescher, Charlotte Lingg, Jan Martin, Bernhard Ulm, Bettina Jungwirth, Manfred Blobner, Stefan J. Schaller

**Affiliations:** ^1^Department of Anesthesiology and Intensive Care, School of Medicine, Klinikum Rechts der Isar, Technical University of Munich, Munich, Germany; ^2^Department of Anesthesiology and Operative Intensive Care Medicine, Charité – Universitätsmedizin Berlin, Corporate Member of Freie Universität Berlin and Humboldt-Universität zu Berlin, Berlin, Germany; ^3^Department of Anesthesiology, Universitätsklinikum Ulm, Ulm, Germany

**Keywords:** frailty, critical illness, outcome assessment, ICU, morbidity

## Abstract

**Background:** Long-term outcome is determined not only by the acute critical illness but increasingly by the reduced functional reserve of pre-existing frailty. The patients with frailty currently account for one-third of the critically ill, resulting in higher mortality. There is evidence of how frailty affects the intrahospital functional trajectory of critically ill patients since prehospital status is often missing.

**Methods:** In this prospective single-center cohort study at two interdisciplinary intensive care units (ICUs) at a university hospital in Germany, the frailty was assessed using the Clinical Frailty Scale (CFS) in the adult patients with critical illness with an ICU stay >24 h. The functional status was assessed using the sum of the subdomains “Mobility” and “Transfer” of the Barthel Index (MTB) at three time points (pre-hospital, ICU discharge, and hospital discharge).

**Results:** We included 1,172 patients with a median age of 75 years, of which 290 patients (25%) were frail. In a propensity score-matched cohort, the probability of MTB deterioration till hospital discharge did not differ in the patients with frailty (odds ratio (*OR*) 1.3 [95% *CI* 0.8–1.9], *p* = 0.301), confirmed in several sensitivity analyses in all the patients and survivors only.

**Conclusion:** The patients with frailty have a reduced functional status. Their intrahospital functional trajectory, however, was not worse than those in non-frail patients, suggesting a rehabilitation potential of function in critically ill patients with frailty.

## Introduction

The number of patients admitted to intensive care units (ICUs) increased within the past years with an ongoing upward trend and an overproportion of the patients advanced in years (1, 2). Older patients are more likely frail, which is a multifaceted condition characterized by the loss of physiologic and cognitive reserves (3, 4). The observational studies suggest that the patients with frailty currently account for up to one-third of the critically ill (5, 6). Consequently, the patient outcome is determined not only by the acute critical illness but increasingly by the reduced functional reserve of pre-existing frailty resulting in higher 30-day mortality (5, 7, 8). In accordance, the likelihood to be discharged to a nursing home is greater in the patients with frailty (9), if the critical illness is survived. This might be caused by the higher odds of disability in the activities in daily living (10, 11) and increased functional dependence (12, 13). Despite this finding, the factors affecting the recovery of physical function after a critical illness remain poorly understood. The patient-level characteristics should be evaluated as the recovery trajectories differ between the cohorts in both the extent and speed of recovery of physical function. A functional trajectory is used to describe this complex process by measuring the changes in the functional status at different time points (14, 15).

While early mobilization might be an important element to maintain the autonomy and mobility in the prior functionally independent patients (16–18), little is known about the functional trajectory of the patients with frailty during the hospital stay (13, 19). Since information about the functional status and mobility of the patients before their ICU admission is typically missing (11, 20), it is unknown if the functional decline is caused by frailty itself, the critical illness, or the combination of both. This might have been important implications for the resource allocations in the acute care setting if the mortality is high and the functional decline cannot be prevented (21, 22).

This study aimed to describe the influence of pre-existing frailty on the functional trajectory of patients with a critical illness during their hospital stay. We hypothesized that the patients with frailty have a greater deterioration of function compared with the patients with non-frailty.

## Materials and Methods

### Study Design, Setting, and Participants

This study is a prospective observational monocentric cohort study of two interdisciplinary ICUs of the Department of Anesthesiology and Intensive Care at Klinikum rechts der Isar, School of Medicine, Technical University of Munich, Germany between April 2017 and May 2019. The Data were extracted from our prospective database of the patients with critical illness who had consented to participate. This prospective analysis was registered at the Clinical Trials and approved by the Ethics Committee of the Faculty of Medicine, Technical University of Munich (528/18 from 22nd Dec 2016). The adults with >24 h stay in the ICU were included, if the consent was obtained either by the patient or legal representative according to the legislation. There were no additional exclusion criteria.

### Outcome Variables

There is no consented outcome measure for the functional status of the patients with critical illness (23). As a substitute, the functional status was therefore recorded with the corresponding subdomains of the Barthel Index, which is an ordinal scale incorporating 10 subdomains of the activities in daily life and the most widely used activities of daily living scale (24, 25). The points of the subdomains “mobility” and “transfer” of the Barthel Index, each ranging between 0 and 15 (“Mobility-Transfer-Barthel”, MTB) were added and represent the functional capacity and gait independence of the patients with a minimum of 0 points (functionally fully dependent) and a maximum of 30 points (functionally independent) (26).

The primary outcome was the probability not to deteriorate in functional status during the hospital stay, i.e., change of the Barthel Score over time, using a baseline value representing the functional status 2 weeks before the hospital admission and at hospital discharge. The prehospital value was obtained retrospectively through the interviews with the patients or their relatives. At ICU and hospital discharge, the functional status was obtained by the study staff. This resulted in a total of three time points to evaluate the individual course of recovery to establish a functional trajectory. The secondary outcome variables were the functional status using the change of MTB till ICU and hospital discharge, the MTB at ICU and hospital discharge, ICU mortality, hospital mortality, ICU length of stay (LOS), and hospital LOS as well as discharge disposition to home.

### Factors

The factor of interest was frailty using the Clinical Frailty Scale (CFS) (5, 27–29). The CFS 9 ranges from 1 “very fit” to 9 “terminally ill” assuming frailty in case of category 5–9 with excellent inter-rater reliability if used in the patients with critical illness (5, 10, 30). The additional factors were age, sex, the Charlson-Comorbidity Index (CCI) (31), and the baseline descriptors at ICU admission, i.e., Sepsis-related Organ Failure Assessment (SOFA) score and Acute Physiology And Chronic Health Evaluation II (APACHE) (32, 33), if the patient was considered neurocritical care (yes/no), and if an elective postoperative admission (yes/no).

### Statistical Analyses

Data analysis was performed with R version 4.0.5 (Austria). The continuous variables were presented as median [interquartile range (IQR)]. The categorical variables were presented using absolute numbers and frequencies.

Propensity matching was performed to balance the influencing factors. A logistic regression modeling was used to calculate the propensity of being frail or non-frail with the factors, such as the patients' age, sex, body mass index (BMI), admission category and department, CCI, as well as SOFA score, APACHE, and Glasgow Coma Scale (GCS) at ICU admission.

The propensity score matching was performed using an R package “Matching” (34). We used a 1:N matching approach with a starting caliper of 0.0001 and repetitive matchings with an increasing caliper (35). After each matching routine, the selected patients with non-frailty were excluded from the further matchings. The procedure was stopped when the necessary sample size was reached to prove the significance (*p* < 0.05) with a power of 80%. The sample size was calculated using the univariate *OR* of 0.524 between all the patients with frailty and non-frailty for the deterioration of the Mobility-Transfer-Barthel till hospital discharge. Assuming a ratio of approximately 1:2 between the patients with frailty and non-frailty, we calculated a necessary total number of 654. With a caliper of 0.0001, 28 patients were selected; with a caliper of 0.001, we obtained a total of 173 patients; with a caliper of 0.01, 483 patients were obtained; and with a caliper of 0.1, we exceeded the necessary threshold obtaining 687 patients. The stepwise resulting subcohorts were not comparable regardless of the caliper chosen when a standardization mean difference (SMD) <0.1 between the groups is required for all the cofactors. The effect sizes of all the endpoints, therefore, were adjusted for these cofactors using multivariate conditional regression.

We performed several sensitivity analyses: first, the primary analysis was repeated in the survivors only in the propensity matched cohort. Second, a logistic regression model for the primary endpoint was used with all the patients. As an exploratory analysis, MTB over time using a multivariate mixed model with the clinically relevant covariates was applied. The independent variables were the same factors as in the propensity score matching, the points in time obtaining the MTB, and the interactions terms of the factor frailty and these points in time. The covariates used were tested for collinearity calculating the variance inflation factor. Missing collinearity was assumed with a variance inflation factor <5; otherwise, one of the factors had to be omitted in the adjusted analysis.

## Results

**Figure 1:**
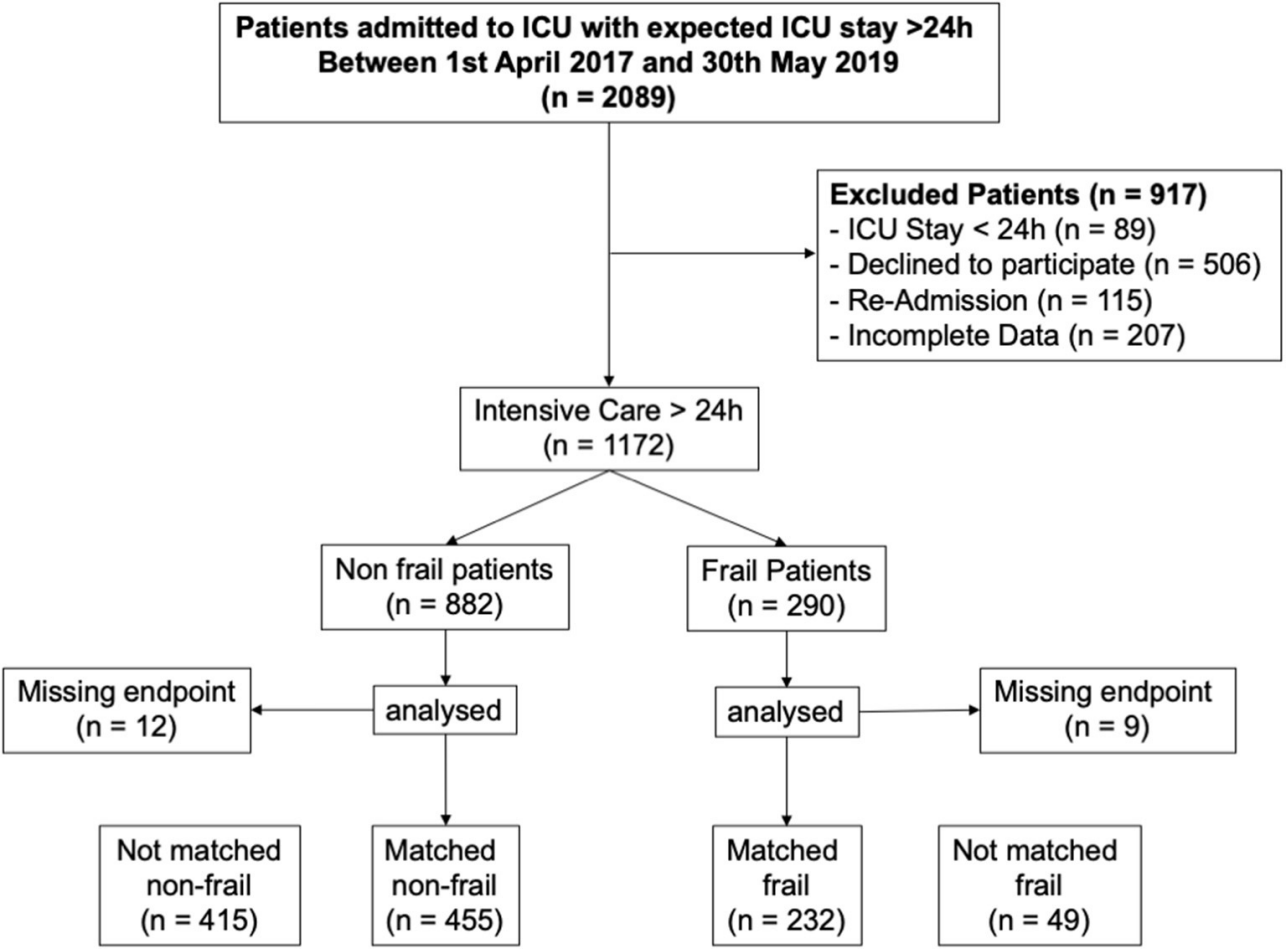
STROBE diagram.

Between April 1, 2017, and May 31, 2019, we included 1,172 patients (Figure 1). The median age was 68 [56–77] years, of which 290 patients (25%) were assessed as frail (CFS levels 5–9). Further patient characteristics are presented in Table 1. Using the propensity score matching, 687 patients were selected, of which 232 were frail and 455 non-frail (Table 2 and Supplementary Table 1 in the Appendix). Applying the same criteria to the survivors, only lead to 393 patients of which 125 were frail (Supplementary Tables 2, 3 in the Appendix).

**Table 1:** The patient characteristics of two interdisciplinary surgical intensive care units (ICUs).

**Patient characteristics**	**Frail (***n*** = 290)**	**Non-frail (***n*** = 882)**
Male^a^	131 (45.2)	332 (37.6)
BMI^a^^,^^b^(kg/m^2^)	25.0 [22.5–27.9]	25.6 [23.3–27.8]
Missing	12 (4.1)	46 (5.2)
Underweight	16 (5.5)	35 (4.0)
Normal	124 (42.8)	347 (39.3)
Overweight	98 (33.8)	324 (36.7)
Obese	40 (13.8)	130 (14.7)
Age (years)^a^	75 [66–82]	65 [54–75]
≤50	19 (6.6)	177 (20.1)
51–65	52 (17.9)	278 (31.5)
66–80	126 (43.4)	324 (36.7)
>80	93 (32.1)	103 (11.7)
Admission from^a^		
Home	170 (58.6)	578 (65.5)
Hospital	97 (33.4)	298 (33.8)
Nursing home	22 (7.6)	1 (0.1)
Unknown	1 (0.3)	5 (0.6)
GCS^a^^,^^b^	13 [5–15]	14 [7–15]
APACHE 2^a^^,^^b^	18 [13–24]	14 [9–19]
SOFA^a^^,^^b^	7 [4.2–10]	6 [4–9]
CCI^a^^,^^b^	3 [1–4]	1 [0–2]
Admission reason^a^		
Sepsis	63 (21.7)	83 (9.4)
Polytrauma	2 (0.7)	38 (4.3)
TBI	20 (6.9)	128 (14.5)
Non-traumatic brain pathology	61 (21.0)	257 (29.1)
Postoperative	49 (16.9)	163 (18.5)
Cardiac	26 (9.0)	49 (5.6)
Pulmonary	119 (41.0)	226 (25.6)
Other	48 (16.6)	134 (15.2)
Department^a^		
Surgical	130 (44.8)	352 (39.9)
Neurological and neurosurgical	124 (42.8)	461 (52.3)
Medical	29 (10.0)	41 (4.6)
Other	7 (2.4)	28 (3.2)

a *Data are n (%) or median [interquartile range (IQR)]*.

b *BMI indicates body mass index; SOFA, Sequential Organ Failure Assessment; APACHE II, Acute Physiology and Chronic Health Evaluation; ICU, intensive care unit; GCS, Glasgow Coma Scale; CCI, Charlson Comorbidity Index; TBI, Traumatic brain injury*.

**Table 2:** Characteristics of the propensity score-matched patients.

	**Not selected**	**Selected**				**Not selected**
	**Non-frail** **(***n*** = 415)**	**Non-frail** **(***n*** = 455)**	**Frail** **(***n*** = 232)**	**SMD**	***p***	**Frail** **(***n*** = 49)**
Male^a^	141 (34.0)	189 (41.5)	98 (42.2)	0.014	0.924	26 (53.1)
BMI (kg/m^2^)^a^^,^^b^	25.4 [23.3–27.8]	25.7 [23.2–28.5]	25.4 [22.7–28.4]	0.017	0.309	24.2 [22.2–27.4]
Missing	14 (3.4)	21 (4.6)	13 (5.6)			3 (6.1)
Underweight	173 (41.7)	170 (37.4)	95 (40.9)			25 (51.0)
Normal	156 (37.6)	162 (35.6)	81 (34.9)			15 (30.6)
Overweight	58 (14.0)	70 (15.4)	37 (15.9)			3 (6.1)
Obese	14 (3.4)	32 (7.0)	6 (2.6)			3 (6.1)
Age (years)^a^	56.0 [43.0–69.0]	71.0 [62.0–78.0]	74.0 [64.8–81.2]	0.170	0.003	80.0 [72.0–84.0]
≤50	145 (34.9)	31 (6.8)	18 (7.8)			1 (2.0)
51–65	140 (33.7)	131 (28.8)	47 (20.3)			4 (8.2)
66–80	104 (25.1)	216 (47.5)	101 (43.5)			20 (40.8)
>80	26 (6.3)	77 (16.9)	66 (28.4)			24 (49.0)
Admission from^a^				0.099	0.619	
Home	275 (66.3)	299 (65.7)	148 (63.8)			15 (30.6)
Hospital	138 (33.3)	152 (33.4)	81 (34.9)			15 (30.6)
Nursing home	0 (0.0)	1 (0.2)	2 (0.9)			19 (38.8)
Unknown	2 (0.5)	3 (0.7)	1 (0.4)			0 (0.0)
GCS^a^^,^^b^	14.0 [7.0–15.0]	14.0 [7.0–15.0]	13.0 [6.0–15.0]	0.079	0.066	9.0 [4.0–15.0]
APACHE 2^a^^,^^b^	11.0 [7.0–17.0]	17.0 [11.0–21.0]	18.0 [12.0–23.2]	0.188	0.038	20.0 [15.0–24.0]
SOFA^a^^,^^b^	6.0 [3.0–8.0]	7.0 [4.0–9.0]	7.0 [4.0–10.0]	0.094	0.228	9.0 [5.0–11.0]
CCI^a^^,^^b^	0.0 [0.0–1.0]	2.0 [0.0–3.0]	2.0 [1.0–4.0]	0.234	<0.001	4.0 [2.0–6.0]
Admission reason^a^^*^						
Sepsis	25 (6.0)	54 (11.9)	49 (21.1)	0.251	0.002	14 (28.6)
Polytrauma	25 (6.0)	13 (2.9)	1 (0.4)	0.192	0.065	1 (2.0)
TBI^b^	70 (16.9)	58 (12.7)	13 (5.6)	0.249	0.005	6 (12.2)
Non-traumatic brain pathology	153 (36.9)	102 (22.4)	48 (20.7)	0.042	0.674	11 (22.4)
Postoperative	73 (17.6)	85 (18.7)	44 (19.0)	0.007	1.000	4 (8.2)
Cardiac	16 (3.9)	33 (7.3)	20 (8.6)	0.051	0.628	3 (6.1)
Pulmonary	79 (19.0)	144 (31.6)	91 (39.2)	0.159	0.058	21 (42.9)
Other	54 (13.0)	78 (17.1)	40 (17.2)	0.003	1.000	7 (14.3)
Specialty^*^				0.078	0.818	
Surgical	133 (32.0)	211 (46.4)	105 (45.3)			22 (44.9)
Neurocritical	255 (61.4)	203 (44.6)	104 (44.8)			18 (36.7)
Medical	11 (2.7)	30 (6.6)	19 (8.2)			7 (14.3)
Other	16 (3.9)	11 (2.4)	4 (1.7)			2 (4.1)

a *Data are n (%)—mean ± SD or median [IQR] SMD—standardized mean difference. Propensity matching was performed with the factors mentioned below in the cohort of all the patients. Reference for sex is male*.

b *BMI indicates body mass index; SOFA, Sequential Organ Failure Assessment; APACHE II, Acute Physiology and Chronic Health Evaluation; ICU, intensive care unit; GCS, Glasgow Coma Scale; CCI, Charlson Comorbidity Index; TBI, Traumatic brain injury*.

* *Not used for matching*.

### Primary Outcome

**Figure 2:**
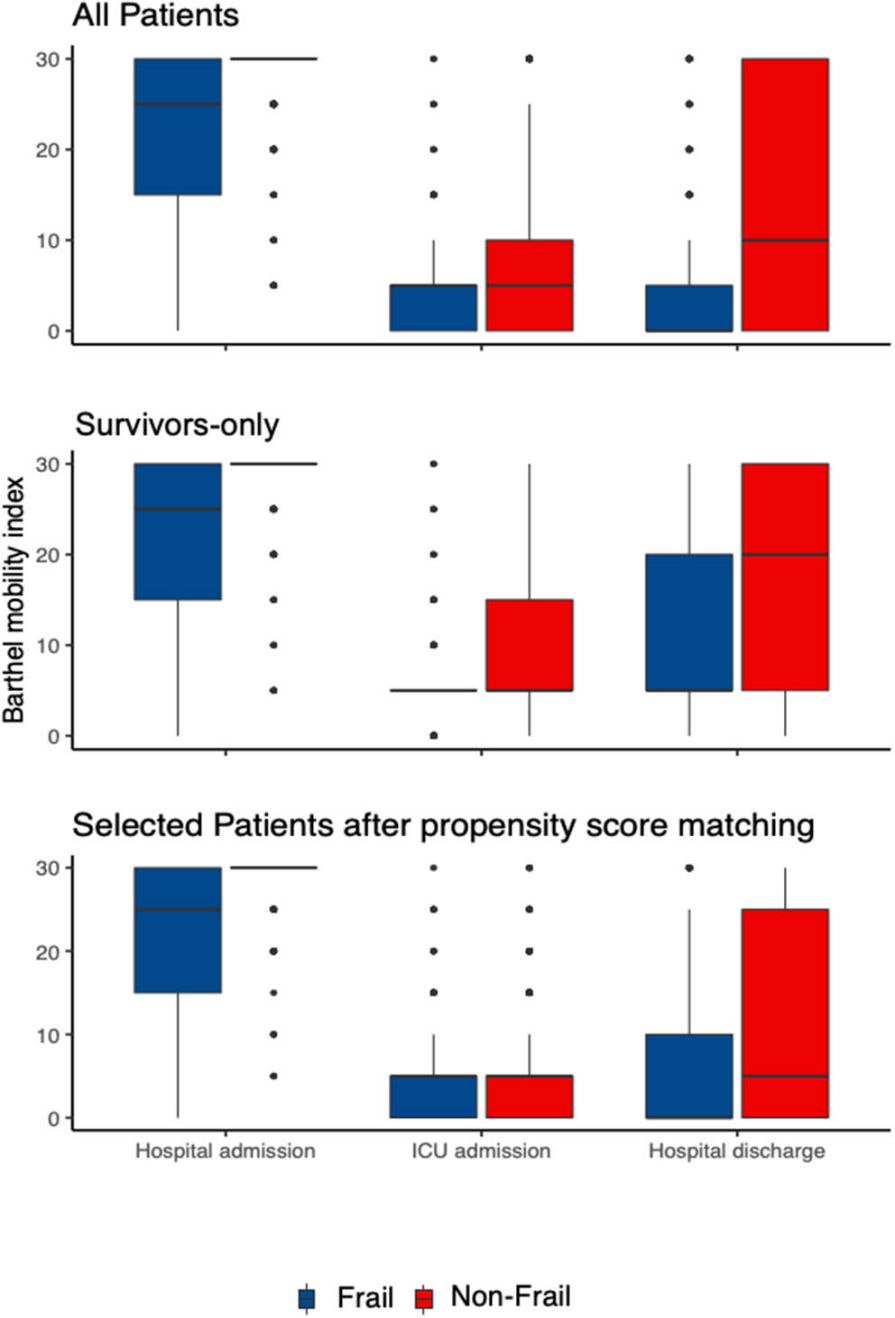
The intrahospital trajectory of the functional status in the patients with frailty vs. non-frailty at three timepoints (“Hospital Admission,” “ICU Discharge,” and “Hospital Discharge”). The functional status is measured as Barthel-Mobility-Index consisting of the two subdomains “Mobility” and “Transfer” of the Barthel-Index, ranging from 0 to 30 (“Mobility-Transfer-Barthel”, MTB). Three patient groups are presented: all patients (*n* = 1,172), survivors only (*n* = 361) and after selection the propensity-matched cohort (*n* = 687). The red boxplots mark the patients with non-frailty and blue the patients with frailty.

Deterioration in MTB occurred in 79% of patients with non-frailty vs. 83% of patients with frailty, an unaltered probability of deterioration in the patients with frailty (*OR* 1.3 [0.8–1.9], *p* < 0.301; Table 3) in the propensity matched cohort (as shown in Figure 2). The sensitivity analysis in that cohort of the survivors revealed similar results (*OR* 1.0 [0.6–1.6], *p* = 1.0), as shown in Supplementary Table 4 in the Appendix. This was confirmed in a further sensitivity analysis using the logistic regression in all the patients (*OR* adj. 0.9 [0.6–1.4], *p* = 0.614), as shown in Figure 2 and Supplementary Table 5 in the Appendix and survivors only (*OR* adj. 1.1 [0.7–1.8], *p* = 0.642), as shown in Figure 2 and Supplementary Table 6 in the Appendix.

**Table 3:** Primary and secondary outcomes in the propensity score matched cohort.

**Variable**	**Non-frail** **patients (***n*** = 455)**	**Frail** **patients (***n*** = 232)**	**Univariate analysis**		**Multivariate analysis**	
			***P*** **-value**	**Effect size**	***P*** **-value**	**Effect size**
**Primary outcome**						
MTB deterioration till hospital discharge	360 (79.1)	192 (82.8)	0.301	1.3 [0.8–1.9]	0.675	1.1 [0.7–1.7]
**Secondary outcome**						
MTB deterioration till ICU discharge	439 (98.0)	203 (90.2)	<0.001	0.2 [0.1–0.4]	<0.001	0.2 [0.1–0.4]
Δ MTB points till ICU discharge	−20 [−30 to −5]	−15 [−25 to −5]	0.012	−5 [−5 to −5]		
Δ MTB points till hospital discharge	−25 [−30 to −20]	−20 [−25 to −10]	<0.001	−5 [−5 to −0]		
ICU length of stay (days)	10 [5–22]	11 [6–23]	0.184	−1 [−2 to 0]		
Hospital length of stay (days)	27 [16–44]	31 [17–47]	0.102	−4 [−6 to 1]		
Mortality (ICU)	99 (21.8)	63 (27.2)	0.139	1.3 [0.9–1.9]	0.663	1.1 [0.7–1.6]
Mortality (hospital)	136 (29.9)	104 (44.8)	<0.001	1.9 [1.4–2.6]	0.003	1.7 [1.2–2.4]
Discharge home	128 (28.1)	32 (13.8)	<0.001	0.4 [0.3–0.6]	0.675	0.4 [0.3–0.7]

*MTB, Mobility-Transfer-Barthel, sum score of the subdomain Mobility and Transfer of the Barthel score*.

### Secondary Outcomes

The probability of MTB deterioration till ICU discharge was significantly reduced in the patients with frailty (*OR* 0.2 [0.1–0.4], *p* < 0.001, Table 3, and Supplementary Tables 4, 7, 8 in the Appendix). The functional trajectory, i.e., the decrease of the MTB till ICU (−20 [95% *CI* −30 to −5] vs. −15 [−25 to −5], *p* < 0.012) and hospital discharge (−25 [−30 to −20] vs. −20 [−25 to −10], *p* ≤ 0.0001) was significantly more pronounced in the patients with non-frailty vs. patients with frailty, respectively (Table 3 and Supplementary Table 4 in the Appendix). ICU and hospital LOS did differ significantly between the patients with frailty and non-frailty in the propensity matched cohort [10 (5–22) vs. 11 (6–23) days, *p* = 0.184 and 27 (16–44) vs. 31 (17–47) days, *p* = 0.102, respectively-Table 3 and Supplementary Table 4 in the Appendix] and in the entire cohort (10 vs. 10 days, *p* = 0.19 and 28 vs. 25 days, *p* = 0.142, respectively-Supplementary Table 9 in the Appendix). This effect could not be validated in the adjusted multivariate analysis. The overall ICU mortality was 29.5% (281/1172). In the propensity matched cohort, there was no difference in the ICU mortality between the non-frail and patients with frailty (22 vs. 27%, *p* = 0.139, Table 3), while there was a significant difference in the complete cohort (18 vs. 30%, *OR* 1.9 [1.4–2.6], *p* < 0.001, Supplementary Table 9 in the Appendix). The results of hospital mortality were similar, with an overall mortality of 48% (361/1,172) and with a significant difference in the propensity matched cohort (30 vs. 44%, *p* ≤ 0.001, Table 3). The patients with non-frailty were discharged home more often (28 vs. 14%, *p* ≤ 0.001, Table 3 and Supplementary Table 9 in the Appendix).

### Exploratory Analysis

Our exploratory analysis using MTB over time confirmed the primary analysis, i.e., the patients with frailty had a lower functional status, however, the decrease of the MTB over time till ICU and hospital discharge was significantly less pronounced in the propensity score cohort (Supplementary Table 10 in the Appendix) and in all the patients (Supplementary Table 11 in the Appendix) and survivors (Supplementary Table 12 in the Appendix).

## Discussion

This prospective observational study refutes the assumption that pre-existing frailty deteriorates the functional status to a greater extent, i.e., the functional trajectory of critical care patients with frailty was not worse compared with the patients with non-frailty when adjusted for age, comorbidity, and the triggering reason for intensive care. Actually, the pre-existing differences in the functional status of patients with frailty and non-frailty converged at hospital discharge, indicating that intensive care is justified in patients with pre-existing frailty as well.

Approximately 25% of the patients were frail when admitted to our ICU for at least 24 h. They had more comorbidities as defined by the CCI, had more insufficient organ systems as indicated by a higher SOFA score, and the overall severity of their diseases was more profound as scored by the APACHE II. Accordingly, the mortality was higher in this patient sub-cohort. These data are in accordance with the German sub analysis of the VIP1 Trial, which included only patients >80 years of age (5). Accounting for more than 50% of patients with frailty indicated a strong relationship between age and reduction of the physiological reserve. Notwithstanding, LOS in the ICU was longer in our cohort when compared with the subcohort of the German VIP Trial (9 vs. 3 days) most likely due to our inclusion criteria of >24 h ICU stay as well as a higher portion of neurocritical care patients.

The patients with frailty expectedly had a reduced functional status during the complete trajectory, i.e., significantly lower MTB. This is in accordance with the other observational studies, which additionally demonstrated increased peri- and postoperative complication rates, morbidity, and mortality (10, 12, 27, 36). Furthermore, Bagshaw et al. suggested that the pre-existing frailty impaired the long-term outcome of ICU survivors, as one-third of their 421 patients reported a reduced health related quality of life with reduced mobility in the physical component score at 6 and 12 months after critical illness (20). In another study in more than 1,000 patients, frailty was again associated with an increased disability after critical illness (11). In both studies, the parameters of functionality were obtained before the onset of critical illness. Brummel et al. investigated the functional status at 3- and 12-month after critical illness, which did not evaluate the influence of intensive care on the clinical outcome. Similarly, in the study from Bagshaw et al., the outcome was assessed at 6 and 12 months. Furthermore, the authors mentioned a limitation that they were not able to integrate the baseline functional measures, such as mobility. Therefore, the conclusion that the functional status deteriorated during intensive care is not justified due to frailty based on those data.

More recently, Ferrante et al. also reported that the patients with frailty ≥70 years old had higher mortality when becoming critically ill (8). Those patients with frailty had an increased disability when compared with the patients with non-frailty. However, the trajectory showed no difference between the patients with frailty and non-frailty. This is similar to our observation in the patients with frailty, whose functional status was also decreased but their trajectory was at least not worse compared with the patients with non-frailty. This also accounts for the long-term outcome study of the same group (37).

The observation of partly improved or unaffected functional status in the patients with frailty who survived their critical illness demands critical consideration. It can be speculated that in older patients, depression, and social isolation often lead to reduced daily activities and accumulation of disability (37). In intensive care, those patients are exposed to stimuli by the caregivers and to early mobilization therapy resulting in the improvement of their functional status (19).

The strength of this study is its prospective approach and the high number of patients included, limited by in-hospital data only. Although this was a single-center study, our cohort showed a heterogeneous group of adult patients regarding diagnosis or prognosis of the disease suggesting generalizability. Assessing the prehospital functional status makes this study unique compared with others and offers new perspectives in understanding the trajectory of critically ill patients with frailty. However, the prehospital frailty status was assessed retrospectively which is a limitation. Although we implemented detailed and recurrent training of our study staff, the assessment depends on the ability to either correctly recall the prehospital status by the patients or to adequately know the status by proxy (memory and information bias). This important problem is not satisfactorily answered yet. The upcoming results of the ASTON study (NCT03785444) will likely improve our insight on assessing the prehospital functional status in patients with critical illness. Until then we must accept this as a limitation. Since the majority of our patients are surgical or trauma, the results should be validated in the medical ICU patients as well.

Addressing the functional outcome is a current focus after surviving the critical illness. Due to the scaling and granularity of the chosen MTB, subtle nuances of the functional outcome might be missed. Since there is no defined core outcome set for the functional outcomes in critical illness yet, we considered the subdomains of the Barthel-Index a suitable option, as it is easy to assess, reproducible for the caregivers, and relevant for the patients (24, 25).

Performing propensity score matching reduced the cohort of the primary analysis considerably. The factors leading to non-selection in propensity scoring were admission from a nursing home, an advanced age, a low GCS, a high APACHE II score, and a high level of comorbidities represented by CCI in the patients with frailty. In the patients with non-frailty, younger age and a low CCI (showing a healthy overall status) lead to non-selection. Nevertheless, this approach strengthens the conclusion that the effect is due to frailty itself and reduces the likelihood of bias. The assumed risk of limited generalizability is countervailed by the confirmation of all the results in the sensitivity and exploratory analysis performed in the complete cohort.

## Conclusion

In conclusion, the patients with frailty have a reduced functional status. Their intrahospital functional trajectory, however, is no worse than those in the patients with non-frailty. Even more, our data suggests a significant rehabilitation potential of functional mobility in the patients with frailty if they survive.

## Data Availability Statement

Data can be obtained from the corresponding author on reasonable scientific request and as long as German data protection law can be complied with. Requests to access the datasets should be directed to Stefan Schaller, stefan.schaller@charite.de.

## Ethics Statement

The studies involving human participants were reviewed and approved by Ethics Committee of the Faculty of Medicine, Technical University of Munich. The patients/participants provided their written informed consent to participate in this study.

## Author Contributions

SJS is the principal investigator and developed the protocol and BU is the study statistician. SJS and MB were involved in the ethical approval. KEF, JJG, BW, RM, BU, BJ, MB, and SJS were involved in the analysis and interpretation of the data. KEF, SF, ML, RB, MH, BK, SK, CL, JM, and SJS were involved in the data acquisition and quality assurance. All authors critically revised the manuscript and approved its final version.

## Conflict of Interest

BW reports personal fees from Orion Pharma Ltd and national (DAAD) and international grants (ESICM) outside the submitted work. BJ received honoraria for giving lectures from Pulsion Medical Systems SE (Feldkirchen, Germany). MB received research support from MSD (Haar, Germany) not related to this manuscript, received honoraria for giving lectures from GE Healthcare (Helsinki, Finland) and Grünenthal (Aachen, Germany). SS reports grants and non-financial support from ESICM (Brussels, Belgium), Fresenius (Germany), Liberate Medical LLC (Crestwood, USA), Reactive Robotics GmbH (Munich, Germany), STIMIT AG (Nidau, Switzerland) as well as from Technical University of Munich, Germany, from national (e.g. DGAI) and international (e.g. ESICM) medical societies (or their congress organizers) in the field of anesthesiology and intensive care, personal fees and non-financial support from Bavarian Medical Association, all outside the submitted work; SS holds stocks in small amounts from Alphabeth Inc., Bayer AG, Rhön-Klinikum AG, and Siemens AG. These did not have any influence on this study. The remaining authors declare that they have no competing interests.

## Publisher's Note

All claims expressed in this article are solely those of the authors and do not necessarily represent those of their affiliated organizations, or those of the publisher, the editors and the reviewers. Any product that may be evaluated in this article, or claim that may be made by its manufacturer, is not guaranteed or endorsed by the publisher.
