# Corrigendum: The functional trajectory in frail compared with non-frail critically ill patients during the hospital stay

**DOI:** 10.3389/fmed.2023.1163166

**Published:** 2023-04-14

**Authors:** K. E. Fuest, Marco Lorenz, Julius J. Grunow, Björn Weiss, Rudolf Mörgeli, Sebastian Finkenzeller, Ralph Bogdanski, Markus Heim, Barbara Kapfer, Silja Kriescher, Charlotte Lingg, Jan Martin, Bernhard Ulm, Bettina Jungwirth, Manfred Blobner, Stefan J. Schaller

**Affiliations:** ^1^Department of Anesthesiology and Intensive Care, School of Medicine, Klinikum Rechts der Isar, Technical University of Munich, Munich, Germany; ^2^Department of Anesthesiology and Operative Intensive Care Medicine, Charité – Universitätsmedizin Berlin, Corporate Member of Freie Universität Berlin and Humboldt-Universität zu Berlin, Berlin, Germany; ^3^Department of Anesthesiology, Universitätsklinikum Ulm, Ulm, Germany

**Keywords:** frailty, critical illness, outcome assessment, ICU, morbidity

In the published article, there was an error in the STROBE diagram as published. In the respective STROBE Diagram, we describe a total of 2089 patients with an expected intensive care stay of >24 h in the period from 1st April 2017 to 30th May 2019. However, the number and differentiation of the excluded patients is not correct.

The corrected Strobe Diagram and its caption appear below.

**Figure 1:**
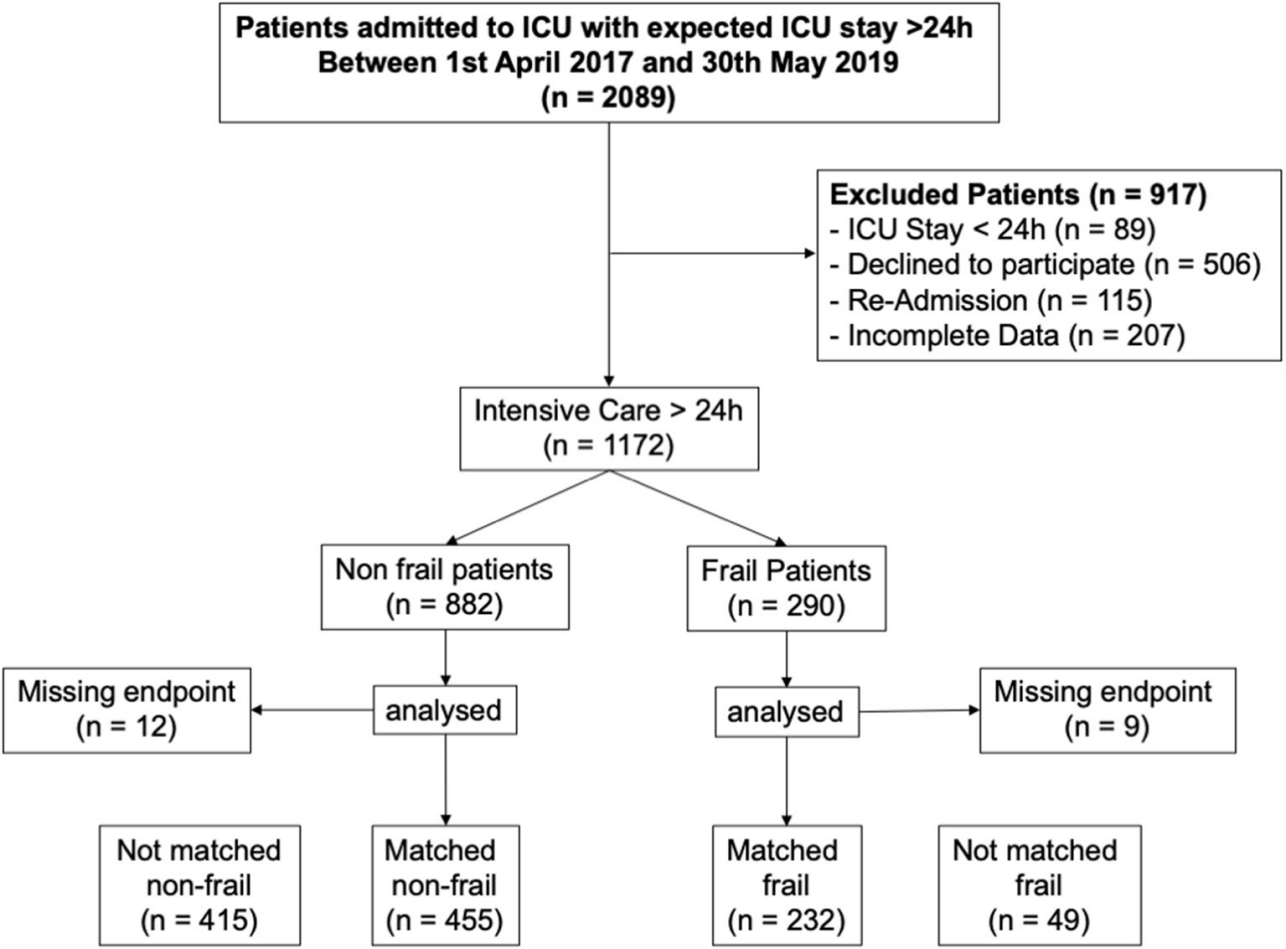
STROBE diagram.

The authors apologize for this error and state that this does not change the scientific conclusions of the article in any way. The original article has been updated.

