# Supplementary material for: The Functional Trajectory in Frail Compared With Non-frail Critically Ill Patients During the Hospital Stay
Source: Front Med (Lausanne). 2021 Nov 4;8:748812. doi: 10.3389/fmed.2021.748812 (PMC8600066; doi:10.3389/fmed.2021.748812)
Supplement: Supplementary file 1 [file Data_Sheet_1.PDF]

Appendix: The functional trajectory of frail compared to non-frail critically-ill patients during the hospital stay

Content of Appendix

Table S1. *Logistic regression model used for propensity score matching* 2
Table S2. *Logistic regression model used for propensity score matching of survivors-only* 3
Table S3. *Characteristics of the propensity score matching in survivors-only* 4
Table S4. *Sensitivity Analysis of the primary and secondary outcomes in the propensity score matched cohort in survivors-only* 5
Table S5. *Deterioration at hospital discharge in a logistic regression model in all patients* 6
Table S6: *Deterioration at hospital discharge in a logistic regression model in survivors-only* 7
Table S7. *Deterioration at ICU discharge in a logistic regression model in all patients.* 8
Table S8. *Deterioration at ICU discharge in a logistic regression model in survivors-only* 9
Table S9. *Secondary outcomes in all patients* 10
Table S10. *Functional trajectory using a mixed model of MTB over time in the propensity score matched cohort* 11
Table S11. *Functional trajectory using a mixed model of MTB over time in all patients* 12
Table S12. *Functional trajectory using a mixed model of MTB over time in survivors-only* 13

Table S1. Logistic regression model used for propensity score matching

	Odds Ratio [95% CI] [a]	P Value
Sex (ref:female)	1.5 [1.1 - 2.0]	0.013
BMI [a] (kg/m^2)	1.0 [1.0 - 1.0]	0.735
Age (years)	1.0 [1.0 - 1.1]	<0.001
Admission from (ref:home)		<0.001
hospital	1.3 [0.9 - 1.7]	0.171
nursing home	43.2 [5.6 - 333.6]	<0.001
unknown	0.6 [0.1 - 5.4]	0.640
GCS [a]	1.0 [1.0 - 1.1]	0.536
APACHE 2 [a]	1.1 [1.0 - 1.1]	<0.001
SOFA [a]	1.0 [0.9 - 1.1]	0.742
CCI [a]	1.2 [1.2 - 1.3]	<0.001
Specialty (ref:Neurocritical)		0.221
Surgical	1.4 [1.0 - 2.0]	0.073
Medical	1.7 [0.9 - 3.2]	0.113
Other	1.1 [0.4 - 2.8]	0.867

This analysis includes all patients, missing values of deceased patients were assigned a value of 0, i.e., the worst possible outcome, Reference for sex is female,
[a] CI: Confidence Interval; BMI: Body Mass Index; SOFA: Sequential Organ Failure Assessment; APACHE II: Acute Physiology and Chronic Health Evaluation; GCS: Glasgow Coma Scale; CCI: Charlson Comorbidity Index

Table S2. Logistic regression model used for propensity score matching of survivors-only

	Odds Ratio [95%CI]	P Value
Sex (ref:female)	1.1 [0.7 - 1.7]	0.605
BMI [a] (kg/m^2) (ref:normal)		0.250
underweight	1.5 [0.7 - 3.5]	0.317
overweight	0.8 [0.5 - 1.2]	0.240
obese	0.7 [0.4 - 1.3]	0.304
missing	0.4 [0.1 - 1.6]	0.180
Age (years) (ref: ≤50)		<0.001
51-65	1.0 [0.5 - 2.1]	0.975
66-80	1.6 [0.8 - 3.3]	0.171
>80	3.6 [1.7 - 7.9]	0.001
Admission from (ref:home)		<0.001
hospital	1.6 [1.0 - 2.4]	0.037
nursing home	43.9 [5.1 - 379.8]	<0.001
unknown	0.0 [0.0 - Inf]	0.979
GCS [a]	1.0 [1.0 - 1.1]	0.354
APACHE 2 [a]	1.1 [1.0 - 1.1]	0.002
SOFA [a]	1.0 [0.9 - 1.1]	0.750
CCI [a]	1.2 [1.1 - 1.3]	<0.001
Specialty (ref:Neurocritical)		0.715
Surgical	1.3 [0.8 - 2.1]	0.249
Medical	1.2 [0.5 - 3.2]	0.709
Other	1.1 [0.4 - 3.1]	0.906

This analysis includes all patients, missing values of expired patients were assigned a value of 0, i.e., the worst possible outcome, Reference for sex is female.

[a] CI: Confidence Interval; BMI: Body Mass Index; SOFA: Sequential Organ Failure Assessment; APACHE II: Acute Physiology and Chronic Health Evaluation; GCS: Glasgow Coma Scale; CCI: Charlson Comorbidity Index

Table S3. Characteristics of the propensity score matching in survivors-only

Characteristics of the propensity score matched survivors

	not selected Non-Frail (n= 384)	selected Non-Frail (n= 268)	Frail (n=125)	SMD	P Value	not selected Frail (n= 21)
Male	153 (39.8)	106 (39.6)	51 (40.8)	0.025	0.901	7 (33.3)
BMI [a] (kg/m2)	25.8 [23.5- 28.4]	24.8 [22.7- 27.7]	24.6 [21.8- 28.5]	0.086	0.716	24.5 [22.8- 27.7]
missing	11 (2.9)	18 (6.7)	10 (8.0)			1 (4.8)
underweight	148 (38.5)	117 (43.7)	57 (45.6)			10 (47.6)
normal	146 (38.0)	87 (32.5)	37 (29.6)			7 (33.3)
overweight	64 (16.7)	39 (14.6)	19 (15.2)			2 (9.5)
obese	15 (3.9)	7 (2.6)	2 (1.6)			1 (4.8)
Age (years)	56.0 [46.0- 68.0]	70.0 [62.0- 77.2]	72.0 [61.0, 78.0]	0.059	0.360	81.0 [75.0- 85.0]
≤50	120 (31.2)	29 (10.8)	13 (10.4)			0 (0.0)
51-65	158 (41.1)	60 (22.4)	29 (23.2)			3 (14.3)
66-80	91 (23.7)	134 (50.0)	58 (46.4)			6 (28.6)
>80	15 (3.9)	45 (16.8)	25 (20.0)			12 (57.1)
Admission from				0.020	0.851	
home	266 (69.3)	172 (64.2)	79 (63.2)			3 (14.3)
hospital	114 (29.7)	96 (35.8)	46 (36.8)			8 (38.1)
nursing home	1 (0.3)	0 (0.0)	0 (0.0)			10 (47.6)
unknown	3 (0.8)	0 (0.0)	0 (0.0)			0 (0.0)
GCS [a]	15 [8- 15]	15 [9- 15]	14 [8- 15]	0.138	0.039	14 [10- 15]
APACHE 2 [a]	11 [7- 16]	15 [10- 19]	15 [11- 21]	0.185	0.163	17 [13- 20]
SOFA [a]	5 [3- 8]	6 [4- 9]	7 [4- 10]	0.101	0.336	6 [4- 8]
CCI [a]	0 [0- 2]	2 [0- 3]	2 [1- 4]	0.258	0.004	3 [1- 4]
Reason for Admission*						
Sepsis	22 (5.7)	29 (10.8)	27 (21.6)	0.296	0.007	7 (33.3)
Polytrauma	28 (7.3)	7 (2.6)	1 (0.8)	0.140	0.423	0 (0.0)
TBI	64 (16.7)	27 (10.1)	9 (7.2)	0.102	0.464	2 (9.5)
Non-traumatic brain pathology	119 (31.0)	54 (20.1)	19 (15.2)	0.130	0.300	2 (9.5)
Postoperative	87 (22.7)	56 (20.9)	33 (26.4)	0.130	0.278	3 (14.3)
Cardiac	9 (2.3)	14 (5.2)	9 (7.2)	0.082	0.585	1 (4.8)
Pulmonary	75 (19.5)	80 (29.9)	51 (40.8)	0.231	0.042	10 (47.6)
Other	57 (14.8)	50 (18.7)	20 (16.0)	0.070	0.617	6 (28.6)
Specialty*				0.084	0.903	
Neurocritical	209 (54.4)	109 (40.7)	52 (41.6)			6 (28.6)
Surgical	149 (38.8)	136 (50.7)	63 (50.4)			10 (47.6)
Medical	11 (2.9)	13 (4.9)	7 (5.6)			3 (14.3)
Other	15 (3.9)	10 (3.7)	3 (2.4)			2 (9.5)

Data are n (%)- mean ± SD or median [IQR] SMD- standardized mean difference. Propensity Matching was performed with the factors mentioned below in the cohort of all patients. Reference for sex is male
[a] BMI: Body Mass Index; SOFA= Sequential Organ Failure Assessment; APACHE II: Acute Physiology and Chronic Health Evaluation; ICU: Intensive Care Unit; GCS: Glasgow Coma Scale; CCI: Charlson Comorbidity Index; TBI: Traumatic Brain Injury. *Not used for matching

Table S4. Sensitivity Analysis of the primary and secondary outcomes in the propensity score matched cohort in survivors-only

	Non-frail patients (n=268)	Frail patients (n=125)	P Value	Effect size	Effect size adjusted for SMD >0.1	Adjusted p for SMD >0.1
	Primary Outcome					
Deterioration until hospital discharge	185 (69.0)	87 (69.6)	1.000	1.0 [0.6 - 1.6]	0.9 [0.6 - 1.5]	0.731
	Secondary Outcome					
Deterioration until ICU discharge	262 (98.1)	108 (86.4)	<0.001	0.1 [0.0 - 0.3]	0.1 [0.0 - 0.3]	<0.001
	∆ Barthel points					
until ICU discharge	-10 [-25, 0]	-10 [-20, 0]	0.123	-5 [-5 - -0]		
until hospital discharge	-25 [-25, -15]	-20 [-25, -5]	<0.001	0 [-5 - 0]		
	Length of Stay (days)					
ICU LOS	11 [4, 23]	10 [5, 24]	0.675	1 [-2 - 1]		
Hospital LOS	32 [20, 50]	36 [21, 53]	0.100	-4 [-8 - 1]		
	Discharge disposition					
Home	112 (41.8)	32 (25.6)	0.003	0.5 [0.3 - 0.8]	0.5 [0.3 - 0.8]	0.005

Table S5. Deterioration at hospital discharge in a logistic regression model in all patients

	Odds Ratio [95%CI] [a]	P Value
Frailty (ref:frail)	0.9 [0.6 - 1.4]	0.614
Sex (ref:female)	1.3 [1.0 - 1.8]	0.097
BMI [a] (kg/m^2) (ref:normal)		0.528
underweight	0.7 [0.3 - 1.3]	0.252
overweight	1.1 [0.8 - 1.6]	0.548
obese	1.3 [0.8 - 2.0]	0.346
missing	1.3 [0.5 - 3.5]	0.563
Age (years) (ref: ≤50)		<0.001
51-65	1.1 [0.7 - 1.8]	0.560
66-80	2.0 [1.2 - 3.2]	0.006
>80	4.7 [2.3 - 9.3]	<0.001
Admission from (ref:home)		0.951
hospital	1.1 [0.8 - 1.5]	0.636
nursing home	1.3 [0.2 - 6.4]	0.783
unknown	1.4 [0.1 - 19.3]	0.793
GCS [a]	1.0 [0.9 - 1.0]	0.517
APACHE 2 [a]	1.0 [1.0 - 1.1]	0.088
SOFA [a]	1.2 [1.1 - 1.2]	<0.001
CCI [a]	1.1 [1.0 - 1.2]	0.025
Specialty (ref:Neurological & Neurosurgical)		<0.001
Surgical	0.3 [0.2 - 0.4]	<0.001
Medical	0.2 [0.1 - 0.4]	<0.001
Other	0.5 [0.2 - 1.4]	0.210
Sepsis (ref: no)	0.9 [0.5 - 1.6]	0.659
Polytrauma (ref: no)	2.4 [0.9 - 6.5]	0.071
Traumatic brain injury (ref: no)	1.1 [0.5 - 2.4]	0.776
Non-traumatic brain pathology (ref: no)	0.9 [0.5 - 1.8]	0.791
Postoperative (ref: no)	0.8 [0.5 - 1.3]	0.360
Cardiac (ref: no)	1.2 [0.5 - 2.8]	0.642
Pulmonary (ref: no)	1.5 [1.0 - 2.5]	0.077
Other (ref: no)	1.1 [0.6 - 2.0]	0.683

This analysis includes all patients, missing values of deceased patients were assigned a value of 0, i.e., the worst possible outcome, Reference for sex is female,

[a] CI: Confidence Interval, BMI: Body Mass Index, SOFA: Sequential Organ Failure Assessment, APACHE II: Acute Physiology and Chronic Health Evaluation, GCS: Glasgow Coma Scale, CCI: Charlson Comorbidity Index

Table S6*:* Deterioration at hospital discharge in a logistic regression model in survivors-only

	Odds Ratio [95% CI] [a]	P Value
Frailty (ref:frail)	1.1 [0.7 - 1.8]	0.642
Sex (ref:female)	1.4 [1.0 - 2.0]	0.070
BMI [a] (kg/m^2) (ref:normal)		0.553
underweight	0.6 [0.3 - 1.3]	0.209
overweight	1.1 [0.8 - 1.6]	0.575
obese	1.2 [0.7 - 2.0]	0.424
missing	1.2 [0.4 - 3.4]	0.745
Age (years) (ref: ≤50)		<0.001
51-65	1.1 [0.7 - 1.8]	0.625
66-80	1.9 [1.1 - 3.2]	0.016
>80	4.4 [2.1 - 9.5]	<0.001
Admission from (ref:home)		0.747
hospital	1.2 [0.8 - 1.7]	0.452
nursing home	1.9 [0.4 - 10.8]	0.446
unknown	1.7 [0.1 - 27.4]	0.708
GCS [a]	1.0 [0.9 - 1.0]	0.380
APACHE 2 [a]	1.0 [1.0 - 1.1]	0.739
SOFA [a]	1.1 [1.1 - 1.2]	<0.001
CCI [a]	1.1 [1.0 - 1.2]	0.116
Specialty (ref:Neurocritical)		<0.001
Surgical	0.3 [0.2 - 0.4]	<0.001
Medical	0.1 [0.1 - 0.4]	<0.001
Other	0.6 [0.2 - 1.7]	0.354
Sepsis (ref:no)	0.9 [0.5 - 1.7]	0.738
Polytrauma (ref:no)	3.2 [1.2 - 8.5]	0.021
Traumatic brain injury (ref:no)	1.2 [0.5 - 2.7]	0.690
Non-traumatic brain pathology (ref:no)	0.8 [0.4 - 1.8]	0.642
Postoperative (ref:no)	0.9 [0.5 - 1.5]	0.683
Cardiac (ref:no)	0.9 [0.4 - 2.3]	0.827
Pulmonary (ref:no)	1.7 [1.0 - 2.8]	0.041
Other (ref:no)	1.1 [0.6 - 2.1]	0.762

Logistic regression model of deterioration at hospital discharge. This analysis includes all survived patients. Reference for sex is female,

[a] CI: Confidence Interval, BMI: Body Mass Index, SOFA: Sequential Organ Failure Assessment, APACHE II: Acute Physiology and Chronic Health Evaluation, GCS: Glasgow Coma Scale, CCI: Charlson Comorbidity Index

Table S7. Deterioration at ICU discharge in a logistic regression model in all patients.

	Odds Ratio [95% CI] [a]	P Value
Frailty (ref:frail)	6.9 [3.2 - 14.9]	<0.001
Sex (ref:female)	1.2 [0.6 - 2.2]	0.606
BMI [a] (kg/m^2) (ref:normal)		0.181
underweight	2.3 [0.5 - 11.1]	0.288
overweight	1.5 [0.8 - 3.0]	0.230
obese	1.3 [0.5 - 3.3]	0.570
missing	4700373.8 [0.0 - Inf]	0.985
Age (years) (ref: ≤50)		0.016
51-65	2.1 [0.9 - 4.8]	0.083
66-80	3.4 [1.4 - 8.4]	0.009
>80	5.3 [1.7 - 16.0]	0.003
Admission from (ref:home)		0.409
hospital	0.6 [0.3 - 1.1]	0.117
nursing home	0.6 [0.1 - 3.3]	0.572
unknown	2295873.7 [0.0 - Inf]	0.995
GCS [a]	1.0 [0.9 - 1.1]	0.766
APACHE 2 [a]	1.0 [0.9 - 1.1]	0.677
SOFA [a]	1.1 [1.0 - 1.3]	0.083
CCI [a]	1.1 [0.9 - 1.3]	0.288
Specialty (ref:Neurocritical)		0.249
Surgical	0.7 [0.3 - 1.9]	0.528
Medical	0.3 [0.1 - 1.1]	0.064
Other	0.3 [0.1 - 1.9]	0.219
Sepsis (ref:no)	0.7 [0.3 - 1.9]	0.481
Polytrauma (ref:no)	1.5 [0.2 - 14.1]	0.712
Traumatic brain injury (ref:no)	1.4 [0.3 - 7.0]	0.651
Non-traumatic brain pathology (ref:no)	0.7 [0.2 - 2.5]	0.628
Postoperative (ref:no)	2.9 [0.8 - 10.2]	0.101
Cardiac (ref:no)	1.7 [0.2 - 15.0]	0.622
Pulmonary (ref:no)	1.6 [0.6 - 4.0]	0.346
Other (ref:no)	0.8 [0.3 - 2.4]	0.736

Logistic regression model of deterioration at ICU discharge. This analysis includes all patients, missing values of deceased patients were assigned a value of 0, i.e., the worst possible outcome, Reference for sex is female,

[a] CI: Confidence Interval; BMI: Body Mass Index; SOFA: Sequential Organ Failure Assessment; APACHE II: Acute Physiology and Chronic Health Evaluation; GCS: Glasgow Coma Scale; CCI: Charlson Comorbidity Index

Table S8. Deterioration at ICU discharge in a logistic regression model in survivors-only

	Odds Ratio [95% CI] [a]	P Value
Frailty (ref:frail)	7.3 [3.2 - 16.9]	<0.001
Sex (ref:female)	1.4 [0.7 - 2.9]	0.332
BMI [a] (kg/m^2) (ref:normal)		0.088
underweight	5.2 [0.6 - 44.4]	0.134
overweight	1.9 [0.9 - 4.1]	0.119
obese	1.3 [0.5 - 3.5]	0.607
missing	3765272.2 [0.0 - Inf]	0.983
Age (years) (ref: ≤50)		0.048
51-65	2.1 [0.9 - 5.3]	0.096
66-80	3.9 [1.4 - 10.7]	0.009
>80	4.2 [1.2 - 14.8]	0.025
Admission from (ref:home)		0.380
hospital	0.6 [0.3 - 1.1]	0.096
nursing home	0.6 [0.1 - 4.0]	0.610
unknown	1430095.1 [0.0 - Inf]	0.995
GCS [a]	1.0 [0.8 - 1.1]	0.572
APACHE 2 [a]	1.0 [0.9 - 1.1]	0.678
SOFA [a]	1.1 [1.0 - 1.3]	0.075
CCI [a]	1.0 [0.8 - 1.2]	0.897
Specialty (ref:Neurocritical)		0.598
Surgical	0.9 [0.3 - 2.6]	0.881
Medical	0.4 [0.1 - 1.6]	0.186
Other	0.8 [0.1 - 7.2]	0.819
Sepsis (ref:no)	0.8 [0.3 - 2.6]	0.736
Polytrauma (ref:no)	1.5 [0.2 - 14.2]	0.707
Traumatic brain injury (ref:no)	1.7 [0.3 - 9.3]	0.557
Non-traumatic brain pathology (ref:no)	0.8 [0.2 - 3.1]	0.762
Postoperative (ref:no)	2.9 [0.8 - 11.0]	0.114
Cardiac (ref:no)	1.2 [0.1 - 11.1]	0.894
Pulmonary (ref:no)	1.7 [0.6 - 4.7]	0.328
Other (ref:no)	1.1 [0.3 - 4.0]	0.865

Logistic regression model of deterioration at ICU discharge. This analysis includes all survivors. Reference for sex is female,

[a] CI: Confidence Interval; BMI: Body Mass Index; SOFA: Sequential Organ Failure Assessment; APACHE II: Acute Physiology and Chronic Health Evaluation; GCS: Glasgow Coma Scale; CCI: Charlson Comorbidity Index

Table S9. Secondary outcomes in all patients

	Non-frail patients (n=870)	Frail patients (n=281)	P Value	Effect size	Effect size adjusted for SMD >0.1	Adjusted p for SMD >0.1
	Primary Outcome					
Deterioration until hospital discharge	638 (73.3)	236 (84.0)	<0.001	1.9 [1.3 - 2.7]	1.3 [0.9 - 1.9]	0.220
	Secondary Outcome					
Deterioration until ICU discharge	827 (97.0)	245 (90.4)	<0.001	0.1 [0.0 - 0.3]	0.1 [0.0 - 0.3]	<0.001
	Δ Barthel points					
until ICU discharge	-20 [-30 - 0]	-15 [-25 - -5]	0.412	-5 [-5 - -5]		
until hospital discharge	-25 [-30 - -20]	-20 [-25 - -10]	<0.001	-5 [-0 - 0]		
	Length of Stay (days)					
ICU LOS	10 [5 - 22]	10 [6 - 22]	0.190	0 [-2 - 0]		
Hospital LOS	25 [15 - 41]	28 [15 - 44]	0.142	-3 [-4 - 1]		
	Mortality					
ICU	159 (18.3)	83 (29.5)	<0.001	1.9 [1.4 - 2.6]	1.2 [0.8 - 1.7]	0.387
Hospital	218 (25.1)	135 (48.0)	<0.001	2.8 [2.1 - 3.7]	1.9 [1.4 - 2.6]	<0.001
	Discharge disposition					
Home	294 (33.8)	35 (12.5)	<0.001	0.3 [0.2 - 0.4]	0.4 [0.3 - 0.6]	<0.001

Table S10. Functional trajectory using a mixed model of MTB over time in the propensity score matched cohort

	Estimate [95% CI] [a]	P Value
Time		<0.001
ICU discharge	-23.3 [-24.2 - -22.4]	<0.001
Hospital discharge	-17.6 [-18.5 - -16.7]	<0.001
Frailty (ref:frail)	-7.2 [-8.4 - -6.0]	<0.001
Sex (ref:female)	-0.5 [-1.3 - 0.3]	0.241
BMI [a] (kg/m^2) (ref:normal)		0.224
underweight	2.0 [0.1 - 3.8]	0.039
overweight	0.5 [-0.4 - 1.4]	0.249
obese	0.3 [-0.9 - 1.5]	0.615
missing	-0.5 [-2.3 - 1.3]	0.581
Age (years) (ref: ≤50)		0.002
51-65	-0.2 [-1.8 - 1.4]	0.798
66-80	-0.7 [-2.3 - 0.9]	0.386
>80	-2.3 [-4.0 - -0.7]	0.006
Admission from (ref:home)		0.039
hospital	-1.2 [-2.0 - -0.4]	0.005
unknown	-1.8 [-7.6 - 4.0]	0.549
GCS [a]	-2.1 [-7.1 - 2.9]	0.417
APACHE 2 [a]	0.1 [-0.1 - 0.2]	0.254
SOFA [a]	-0.1 [-0.2 - -0.0]	0.032
CCI [a]		<0.001
Specialty (ref:Neurocritical)	-0.2 [-0.3 - -0.0]	0.031
Surgical	-0.2 [-0.4 - -0.0]	0.019
Medical	3.2 [2.1 - 4.4]	<0.001
Other	1.7 [-0.1 - 3.5]	0.065
Sepsis (ref:no)	6.0 [3.3 - 8.8]	<0.001
Polytrauma (ref:no)	-0.4 [-1.7 - 0.9]	0.503
Traumatic brain injury (ref:no)	-1.5 [-4.3 - 1.4]	0.312
Non-traumatic brain pathology (ref:no)	0.7 [-1.0 - 2.5]	0.417
Postoperative (ref:no)	-0.0 [-1.5 n= 1.5]	0.983
Cardiac (ref:no)	1.2 [-0.1 n= 2.5]	0.075
Pulmonary (ref:no)	-0.9 [-2.5 n= 0.7]	0.292
Other (ref:no)	0.1 [-1.0 n= 1.2]	0.830
Interaction Time Frailty		<0.001
ICU [a] discharge*Non-Frail	-0.4 [-1.8 n= 1.1]	0.608
Hospital discharge*Non-Frail	5.8 [4.2 n= 7.4]	<0.001

This analysis includes all selected patients, missing values of deceased patients were assigned a value of 0, i.e., the worst possible outcome, reference for sex is female.

[a] CI: Confidence Interval; BMI: Body Mass Index; SOFA: Sequential Organ Failure Assessment; APACHE II: Acute Physiology and Chronic Health Evaluation; GCS: Glasgow Coma Scale; CCI: Charlson Comorbidity Index; ICU: Intensive Care Unit; MTB: Mobility-Transfer-Barthel, sum score of the subdomain Mobility and Transfer of the Barthel Score

Table S11. Functional trajectory using a mixed model of MTB over time in all patients

	Estimate [95% CI] [a]	P Value
Time		<0.001
ICU discharge	-22.9 [-23.6 n= -22.2]	<0.001
Hospital discharge	-16.4 [-17.1 n= -15.7]	<0.001
Frailty (ref:frail)	-6.4 [-7.5 n= -5.3]	<0.001
Sex (ref:female)	-0.4 [-1.0 n= 0.2]	0.209
BMI [a] (kg/m^2) (ref:normal)		0.320
underweight	1.0 [-0.5 n= 2.6]	0.181
overweight	0.5 [-0.2 n= 1.2]	0.172
obese	0.4 [-0.6 n= 1.3]	0.448
missing	-0.7 [-2.2 n= 0.8]	0.359
Age (years) (ref: ≤50)		<0.001
51-65	-0.8 [-1.7 n= 0.2]	0.104
66-80	-1.6 [-2.6 n= -0.6]	0.001
>80	-3.2 [-4.3 n= -2.0]	<0.001
Admission from (ref:home)		0.075
hospital	-0.9 [-1.6 n= -0.2]	0.010
unknown	-0.6 [-2.9 n= 1.7]	0.610
GCS [a]	-1.2 [-5.3 n= 3.0]	0.584
APACHE 2 [a]	0.1 [-0.0 n= 0.2]	0.176
SOFA [a]	-0.1 [-0.2 n= -0.0]	0.002
CCI [a]		<0.001
Specialty (ref:Neurocritical)	-0.3 [-0.4 n= -0.1]	<0.001
Surgical	-0.2 [-0.3 n= -0.0]	0.012
Medical	3.3 [2.4 n= 4.2]	<0.001
Other	2.7 [1.2 n= 4.1]	<0.001
Sepsis (ref:no)	5.1 [3.2 n= 6.9]	<0.001
Polytrauma (ref:no)	-1.0 [-2.1 n= 0.1]	0.085
Traumatic brain injury (ref:no)	-1.2 [-2.9 n= 0.6]	0.197
Non-traumatic brain pathology (ref:no)	0.3 [-1.0 n= 1.6]	0.688
Postoperative (ref:no)	-0.2 [-1.4 n= 1.0]	0.727
Cardiac (ref:no)	0.7 [-0.3 n= 1.8]	0.155
Pulmonary (ref:no)	-0.4 [-1.8 n= 1.0]	0.548
Other (ref:no)	-0.2 [-1.1 n= 0.6]	0.586
Interaction Time Frailty		<0.001
ICU [a] discharge*Non-Frail	-0.6 [-1.8 n= 0.6]	0.334
Hospital discharge*Non-Frail	5.2 [3.8 - 6.6]	<0.001

This analysis includes all patients, missing values of expired patients were assigned a value of 0, i.e., the worst possible outcome, reference for sex is female,

[a] CI: Confidence Interval; BMI: Body Mass Index; SOFA: Sequential Organ Failure Assessment; APACHE II: Acute Physiology and Chronic Health Evaluation; GCS: Glasgow Coma Scale; CCI: Charlson Comorbidity Index; ICU: Intensive Care Unit; MTB: Mobility-Transfer-Barthel, sum score of the subdomain Mobility and Transfer of the Barthel Score

Table S12. Functional trajectory using a mixed model of MTB over time in survivors-only

	Estimate [95% CI] [a]	P Value
Time		<0.001
ICU discharge	-21.2 [-22.0 - -20.5]	<0.001
Hospital discharge	-12.0 [-12.8 - -11.3]	<0.001
Frailty (ref:frail)	-7.1 [-8.5 - -5.6]	<0.001
Sex (ref:female)	-0.5 [-1.3 - 0.2]	0.174
BMI [a] (kg/m^2) (ref:normal)		0.407
underweight	1.2 [-0.5 - 2.9]	0.177
overweight	0.6 [-0.2 - 1.5]	0.140
obese	0.7 [-0.4 - 1.8]	0.224
missing	-0.3 [-2.4 - 1.9]	0.815
Age (years) (ref: ≤50)		0.003
51-65	-0.8 [-1.9 - 0.3]	0.159
66-80	-1.2 [-2.4 - -0.1]	0.038
>80	-2.8 [-4.2 - -1.3]	<0.001
Admission from (ref:home)		0.038
hospital	-1.1 [-1.9 - -0.3]	0.011
unknown	-2.7 [-5.9 - 0.6]	0.105
GCS [a]	-0.7 [-6.6 - 5.2]	0.814
APACHE 2 [a]	0.1 [-0.1 - 0.2]	0.258
SOFA [a]	-0.1 [-0.2 - 0.0]	0.083
CCI [a]		<0.001
Specialty (ref:Neurocritical)	-0.2 [-0.4 - -0.1]	0.006
Surgical	-0.2 [-0.3 - 0.0]	0.102
Medical	4.0 [2.9 - 5.1]	<0.001
Other	4.4 [2.4 - 6.4]	<0.001
Sepsis (ref:no)	4.8 [2.8 - 6.9]	<0.001
Polytrauma (ref:no)	-0.9 [-2.3 - 0.5]	0.213
Traumatic brain injury (ref:no)	-2.3 [-4.2 - -0.4]	0.018
Non-traumatic brain pathology (ref:no)	0.4 [-1.3 - 2.0]	0.666
Postoperative (ref:no)	0.7 [-0.8 - 2.2]	0.366
Cardiac (ref:no)	0.2 [-1.0 - 1.4]	0.701
Pulmonary (ref:no)	0.6 [-1.3 - 2.6]	0.515
Other (ref:no)	-0.2 [-1.3 - 0.9]	0.698
Interaction Time Frailty		<0.001
ICU [a] discharge*Non-Frail	-0.6 [-2.1 - 0.8]	0.393
Hospital discharge*Non-Frail	6.0 [4.2 - 7.8]	<0.001

This analysis includes all survived patients, missing values of expired patients were assigned a value of 0, i.e., the worst possible outcome, reference for sex is female,

[a] CI: Confidence Interval; BMI: Body Mass Index; SOFA: Sequential Organ Failure Assessment; APACHE II: Acute Physiology and Chronic Health Evaluation; GCS: Glasgow Coma Scale; CCI: Charlson Comorbidity Index; ICU: Intensive Care Unit; MTB: Mobility-Transfer-Barthel, sum score of the subdomain Mobility and Transfer of the Barthel Score
